# The importance of high quality real-life social interactions during the COVID-19 pandemic

**DOI:** 10.1038/s41598-023-30803-9

**Published:** 2023-03-04

**Authors:** Maximilian Monninger, Pascal-M. Aggensteiner, Tania M. Pollok, Anna Kaiser, Iris Reinhard, Andrea Hermann, Markus Reichert, Ulrich W. Ebner-Priemer, Andreas Meyer-Lindenberg, Daniel Brandeis, Tobias Banaschewski, Nathalie E. Holz

**Affiliations:** 1 Department of Child and Adolescent Psychiatry and Psychotherapy, Central Institute of Mental Health, Medical Faculty Mannheim/Heidelberg University, J5, 68159 Mannheim, Germany; 2 Department of Biostatistics, Central Institute of Mental Health, Medical Faculty Mannheim/Heidelberg University, J5, 68159 Mannheim, Germany; 3 Department of Psychotherapy and Systems Neuroscience, Justus Liebig University, Giessen, Germany; 4 Bender Institute of Neuroimaging, Justus Liebig University, Giessen, Germany; 5 Center for Mind, Brain and Behavior, Phillips University Marburg and Justus Liebig University, Giessen, Germany; 6 Department of Psychiatry and Psychotherapy, Central Institute of Mental Health, Medical Faculty Mannheim/Heidelberg University, J5, 68159 Mannheim, Germany; 7 Mental mHealth Lab, Institute of Sport and Sports Science, Karlsruhe Institute of Technology, Engler-Bunte Ring 15, 76131 Karlsruhe, Germany; 8 Department of Child and Adolescent Psychiatry and Psychotherapy, Psychiatric University Hospital Zurich, University of Zurich, Neumünsterallee 9, 8032 Zurich, Switzerland; 9 Neuroscience Center Zurich, University of Zurich and ETH Zurich, Winterthurerstr. 190, 8057 Zurich, Switzerland; 10 Donders Institute, Radboud University, Nijmegen, The Netherlands; 11 Radboud University Medical Centre, Nijmegen, The Netherlands; 12 Institute of Medical Psychology and Medical Sociology, University Medical Center Schleswig Holstein, Kiel University, Kiel, Germany; 13 Donders Centre for Cognitive Neuroimaging, Donders Institute for Brain, Cognition and Behaviour, Radboud University, Kapittelweg 29, 6525 EN Nijmegen, The Netherlands

**Keywords:** Neuroscience, Psychology

## Abstract

The coronavirus pandemic has brought about dramatic restrictions to real-life social interactions and a shift towards more online social encounters. Positive social interactions have been highlighted as an important protective factor, with previous studies suggesting an involvement of the amygdala in the relationship between social embeddedness and well-being. The present study investigated the effect of the quality of real-life and online social interactions on mood, and explored whether this association is affected by an individual’s amygdala activity. Sixty-two participants of a longitudinal study took part in a one-week ecological momentary assessment (EMA) during the first lockdown, reporting their momentary well-being and their engagement in real-life and online social interactions eight times per day (N ~ 3000 observations). Amygdala activity was assessed before the pandemic during an emotion-processing task. Mixed models were calculated to estimate the association between social interactions and well-being, including two-way interactions to test for the moderating effect of amygdala activity. We found a positive relationship between real-life interactions and momentary well-being. In contrast, online interactions had no effect on well-being. Moreover, positive real-life social interactions augmented this social affective benefit, especially in individuals with higher amygdala being more sensitive to the interaction quality. Our findings demonstrate a mood-lifting effect of positive real-life social interactions during the pandemic, which was dependent on amygdala activity before the pandemic. As no corresponding effect was found between online social interactions and well-being, it can be concluded that increased online social interactions may not compensate for the absence of real-life social interactions.

## Introduction

The coronavirus (COVID-19) pandemic brought about dramatic changes to everyday life for hundreds of millions of people around the world. With the social contact restrictions beginning in March 2020 in Germany, nearly all schools were closed overnight, working environments changed radically, and social life was governmentally restricted like never before^1^. A variety of population-based studies around the globe have reported elevated levels of perceived stress, increased feelings of loneliness, and a rise of anxiety and depressive symptoms during lockdowns, and studies with clinical samples have highlighted symptom deteriorations^2–6^.

It is widely reported that social contacts and social networks are important for subjective well-being^7,8^ as well as psychological and physical health^9–14^. While these findings point to a positive relationship for social network sizes and overall well-being, conceptualization of these studies might be problematic. Most of these studies used retrospectively collected single measures of social network characteristics (i.e. the social network size or the amount of Facebook friends) and overall well-being^15,16^, capturing between-subject associations. However, these approaches fail to carefully identify time-dependent short-term alterations in well-being evoked by social interactions on a within-subject level.

Just recently, the relationship between social interactions and momentary well-being has addressed using ecological momentary assessment (EMA)^17–20^. Findings from these studies consistently indicate that physically being in the company strongly predicts higher levels of momentary well-being and positive mood^17–19^. However, little is known about how specific characteristics of social interactions (i.e., the social interaction partner, the self-perceived quality of the social interaction) are related to momentary well-being. Importantly, the effects of these momentary and highly volatile mood states, thereby capturing within-person associations, have been demonstrated to be associated with between-subject measures of overall well-being, resilience, and mental health^18,21^. For instance, Reichert et al. found that participants with higher levels of momentary energetic arousal as an indicator of positive mood (within-person construct) reported elevated levels of overall well-being, satisfaction with life, or optimism (between-person constructs), indicating a robust relationship between moment-level variables and person-level variables^21^. Research regarding the effects of online interactions, by contrast, remains scarce^22^. While a variety of cross-sectional studies have reported small and often heterogeneous associations between the average time spent on online social media platforms and decreased well-being and mood^23–25^, studies exploring the real-time and individual effects of online interactions on mood are sparse. For instance, Beyens et al. investigated the association between different social media platforms (e.g. WhatsApp, Facebook, Instagram) and well-being in a sample of adolescents using EMA^26^. On a between-person level, time spent on social media was not related to affective well-being. However, when focusing on within-person effects, around 10% of the sample reported lower levels of well-being when passively using social media, whereas almost equally participants had either higher levels of well-being or showed no significant association between social media use and well-being, respectively^26^. Just recently, a large-scale study reported the momentary affective benefit of real-life over online interaction in adolescents^27^. Interestingly, adolescents showed an affective benefit from digital contacts, which, however, was smaller compared to the affective benefit from being physically in company with others^27^. Taken together, the association between online interactions and well-being has been reported to be highly heterogeneous on an individual level, which might be diluted in group averages, resulting in a need for within-person approaches.

In addition, the reported studies focused on social-media usage in adolescents, predominantly capturing usage frequency and time spent online, thereby neglecting real-life social contacts. Moreover, these studies were performed during normal times, when access to face-to-face interactions was unrestrained. Given that real-life interactions were restricted during the initial lockdown phase of the pandemic, and a shift towards more online social interactions was observed^28^, it is important to determine whether this enforced change led to an individual social affective benefit that may have compensated for the lack of social interactions in real life.

From a neural perspective, the volume and function of the amygdala have been consistently shown to be related to an individual’s social network size^16,29,30^. Indeed, the amygdala has been recognized as a key region of the social brain^31^, with its particular involvement in social adaption and its high susceptibility to socioenvironmental influences^32^. Recently, it has been demonstrated that brain volume in the limbic circuit, which was previously linked to social affective benefit and social stress processing^31^, modulates the real-time affective benefit of being in company^19^, suggesting a possible involvement of neural markers on moment-level variables.

However, little is known about how neural activity might influence this social affective benefit in daily life. One pathway could be that the immediate evaluation of the quality of a social interaction might be moderated by an individual’s neural activity (i.e. the amygdala activation), thereby affecting the individual´s perception of the interaction, which may, in turn, result in a mood-lifting or mood-worsening effect. However, this association has not yet been addressed either in a real-time, real-life setting or during times of crisis when access to social support was drastically limited but particularly crucial^33^. Accordingly, by combining EMA data with neural activity, we will be able to exploratory investigate this potential pathway in the relationship between social interactions, neural mechanisms and well-being.

The present study therefore investigated the impact of real-life and online interactions on well-being in a naturalistic setting during the first COVID-19 pandemic-related lockdown in Germany.

Previous studies mostly focused on between-person designs, which per nature might lead to heterogeneous findings with limited generalizability to the individual level^34,35^. With regard to this ‘ecological fallacy’^36,37^ it is highly important to choose between within- and between-subject approaches depending on the research question^37^. For instance, while between-subject designs are appropriate for examining specific risk profiles, within-subject designs are suitable to detect individual associations over time^37^. Given our extensive longitudinal assessment, we specifically tackled the social affective benefit at the individual level, using a within-person approach to investigate how momentary well-being is influenced by real-life and online interactions and their specific characteristics, while only controlling for time invariant predictors (between-subject factors, e.g., sex, psychosocial risk). While we hypothesized a social benefit after real-life social interactions^19^, inconsistent prior findings hamper drawing a specific a-priori hypothesis with regard to online interactions. However, in view of a pandemic-evoked shift towards more online communication and a lack of real-life contacts due to restrictions, we hypothesized that participation in online interactions would predict a similar affective benefit to real-life interactions (Model I). Next, we attempted to differentiate the impact of distinct characteristics of social interactions (i.e., quality of social interactions, interaction partner, and liking of interaction partner) on well-being. As structural characteristics (i.e. the objective quantity of social interactions) were disrupted due to the social contact restrictions during lockdown^19,38^ to a greater extent than subjective characteristics of social relationships (i.e. the self-perceived quality of a social interaction)^38^, we expected the quality of social interactions to be particularly important during the initial lockdown (Model II). Finally, we exploratory investigated the possible moderating effect of amygdala activity, assessed before the COVID-19 pandemic, on the association between the characteristics of social interactions and perceived well-being. Based on previous findings^16^, we expected a stronger relationship between social interactions and well-being in those participants with heightened amygdala activity due to their possibly increased awareness of social cues (Model III).

## Results

### Descriptive data

Demographic data are depicted in Table 1. On average, participants (N = 62) answered a total of 48.1 EMA prompts (SD = 8.7; range = 10–56) during 1 week (7 days), resulting in a high compliance rate of 85.80%. The earliest starting day of the EMA week was 4.6 weeks (32 days) after the onset of the social contact restrictions in Germany on 22nd March 2020. For 2,556 prompts (84.6%), participants reported engagement in real-life social interactions, while online interactions were reported on 1851 occasions (61.3%). On 1,900 occasions (74.3% of all real-life interactions), participants reported the most important real-life interaction partner to be a family member, whereas online contacts were predominantly with non-family members (578 situations, 31.2%). Liking of the most important interaction partner was rated as very high overall (real-life contacts: mean = 88.3; SD = 15.6; range: 0–100; online contacts: mean = 80. 5; SD = 15.4; range = 0–100). The quality of the most important interactions was positively rated overall (real-life: mean = 75.9; SD = 17.9; range = 0–100; online: mean = 72.3; SD = 17.3; range = 0–100).

**Table 1:** Sample characteristics and descriptive data.

Descriptive data (n = 62)			
	N	%	
Gender (female)	36	58.06	
Critical worker status	24	38.71	
Full-time employment	37	59.68	
Workplace changes due to COVID-19	39	62.90	
Parenthood	27	38.70	

	Mean	SD	Range
Well-being (positive affect)	4.55	0.97	1–7
Household members	2.74	1.24	1–6
Age (years)	33.33	0.58	32.250–34.25
Psychosocial risk factors at birth	1.9	1.9	0–

### Social contacts and well-being (Model I)

**Figure 1:**
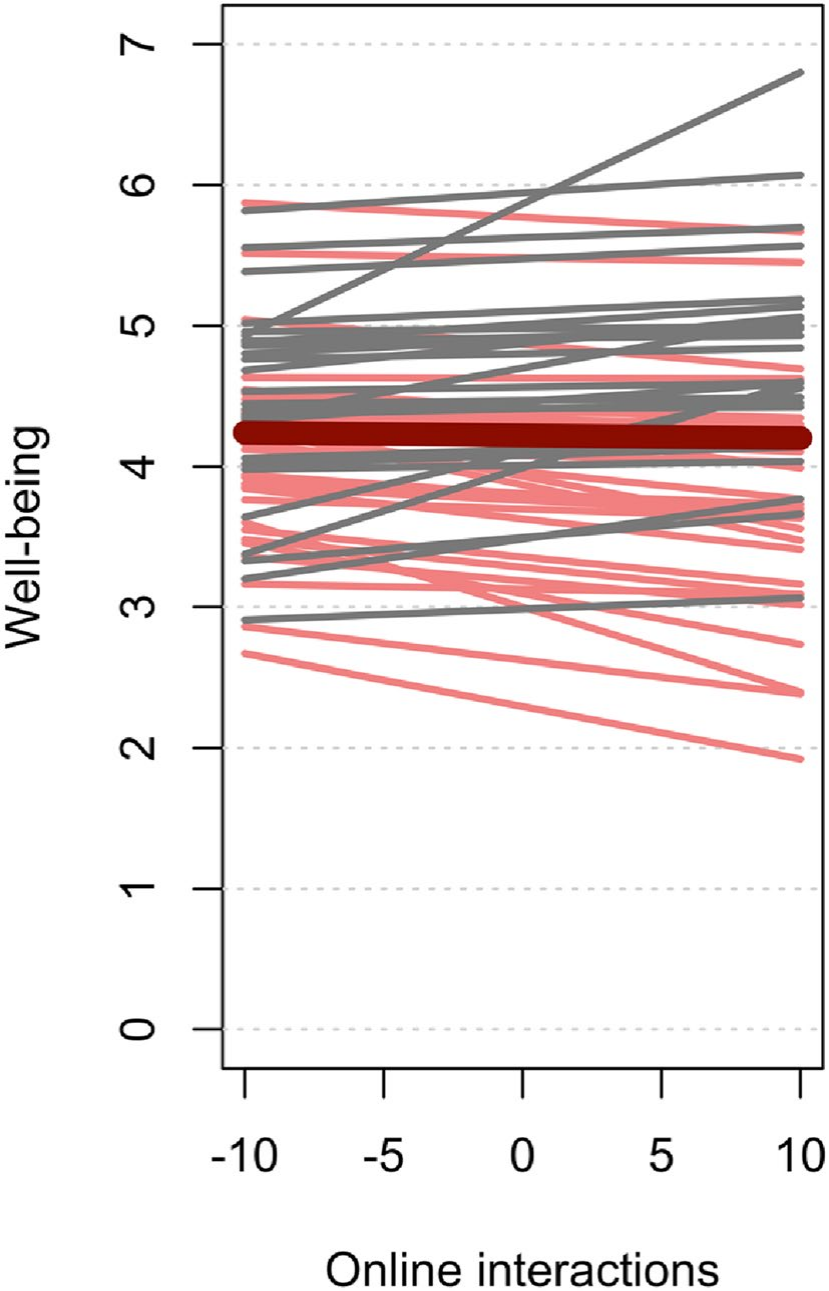
Association of online social interactions and affective well-being, indicating that the amount of online social interactions did not predict well-being. Online social interactions are person-mean centered and differences from zero indicate a lower or higher number of interactions compared to the person mean.

Initially, we investigated whether the presence of real-life and online social interactions was associated with well-being. Therefore, we calculated multilevel models with EMA data of current well-being as dependent variable and dimensions of social interactions as predictor variables (level 1) nested within participants (level 2). In addition, psychosocial risk factors at birth, gender, time of day, critical worker status (yes/no), and number of weeks since social contact restrictions began (22^nd^ March 2020) were included as covariates of no interest. We found that well-being was positively associated with the presence (in contrast to being alone) of real-life social interactions during the social contact restrictions in Germany (β = 0.084, SE = 0.038, T_(2929.75)_ = 5.924, p < 0.001, Table 2), whereas no significant association was found for engagement in online interactions (β = 0.013, SE = 0.027, T_(2909.85)_ = 0.967, p = 0.333). There were also no significant associations with any of the covariates. This relationship remained unchanged after including time-lagged well-being as an additional predictor of no interest (β = 0.015, SE = 0.025, T_(2887.58)_ = 4.786, p < 0.001). As online interactions were not related to well-being (Fig. 1), the main analyses focused on characteristics of real-life social interactions. However, all results for characteristics of online interactions are outlined in the results section of the Supplementary Information.

**Table 2:** Mixed model effects of presence of real-life and online social interactions on well-being.

Predictors	Well-being				
	Estimates	Std. Beta	CI	Standardized CI	p
(Intercept)	4.2018	− 0.1423	3.6486 – 4.7550	− 0.4438 – 0.1592	< 0.001
Gender	0.1637	0.1686	− 0.2090 – 0.5363	− 0.2153 – 0.5524	0.389
Psychosocial risk factors at birth	− 0.0674	− 0.1316	− 0.1655 – 0.0308	− 0.3233 – 0.0602	0.179
Time of day	0.0018	0.0085	− 0.0068 – 0.0105	− 0.0316 – 0.0486	0.678
Critical worker status	0.2256	0.2323	− 0.1531 – 0.6042	− 0.1576 – 0.6223	0.243
Presence of real-life social interactions (yes / being alone)	0.2268	0.0838	0.1517 – 0.3018	0.0561 – 0.1116	** < 0.001**
Presence of online interactions (yes/ no)	0.0261	0.0131	− 0.0268 – 0.0789	− 0.0134 – 0.0396	0.333
Weeks since lockdown	− 0.0014	− 0.0023	− 0.0686 – 0.0657	− 0.1115 – 0.1069	0.967
Random effects					
σ^2^	0.4094				
τ_00_ _Participants_	0.5865				
τ_11_ _Time of day_	0.0008				
ICC	0.57				
N	62				
Observations	2971				
Marginal R^2^/Conditional R^2^	0.037/0.585				

Significant values are in bold.

### Quality of social interactions and well-being (Model II)

In a second step, we explored whether the quality of a social interaction, the interaction partner, and the liking of the most important interaction partner were related to well-being. In situations in which real-life social interactions were reported, a positive association between the quality of social interactions and well-being was observed (β = 0.214, SE = 0.001, T_(2459.40)_ = 15.424, p < 0.001, Table 3). We found no significant effect for the most important interaction partner (β = -0.059, SE = 0.041, T_(2512.54)_ = -1.373, p = 0.170) or liking of the most important interaction partner on well-being (β = 0.011, SE = 0.001, T_(2472.52)_ = 0.718, p = 0.477). This association remained significant after including time-lagged well-being as an additional predictor of no interest (β = 0.182, SE = 0.001, T_(2411.32)_ = 13.746, p < 0.001).

**Table 3:** Mixed model effects of distinct characteristics of real-life social interactions on well-being.

Predictors	Well-being				
	Estimates	Std. Beta	CI	Standardized CI	p
(Intercept)	4.6593	− 0.1284	4.1015 – 5.2171	− 0.4450 – 0.1882	< 0.001
Gender	0.1805	0.1918	− 0.1910 – 0.5520	− 0.2030 – 0.5865	0.341
Psychosocial risk factors at birth	− 0.0655	− 0.1328	− 0.1635 – 0.0324	− 0.3313 – 0.0656	0.190
Time of day	− 0.0031	− 0.0147	− 0.0109 – 0.0047	− 0.0518 – 0.0225	0.439
Critical worker status	0.2210	0.2348	− 0.1566 – 0.5986	− 0.1664 – 0.6359	0.251
Most important interaction partner	− 0.0563	− 0.0598	− 0.1366 – 0.0240	− 0.1452 – 0.0255	0.170
Quality of the relationship with the most important interaction partner	0.0008	0.0119	− 0.0015 – 0.0032	− 0.0210 – 0.0448	0.477
COVID-19-related content of the most important interaction	0.0008	0.0168	− 0.0004 – 0.0020	− 0.0094 – 0.0430	0.210
Quality of the most important interaction	0.0138	0.2141	0.0121 – 0.0156	0.1869 – 0.2413	** < 0.001**
Weeks since lockdown	− 0.0213	− 0.0355	− 0.0896 – 0.0469	− 0.1492 – 0.0782	0.540
Random effects					
σ^2^	0.3623				
τ_00_ _Participants_	0.5591				
τ_11_ _Time of day_	0.0005				
ICC	0.59				
N	62				
Observations	2537				
Marginal R^2^ /Conditional R^2^	0.074/0.624				

Significant values are in bold.

Additional, Granger causality tests were performed to analyze a possible causal relationship between quality of the most important interaction and well-being and vice versa. Findings indicate that the perceived quality is predictive of well-being in the future (F_(2569)_ = 5.11, p = 0.024), whereas the opposite direction did not reach significance (F_(2569)_ = 2.54, p = 0.111).

### Amygdala activity, social interactions, and well-being (Model III)

**Figure 2:**
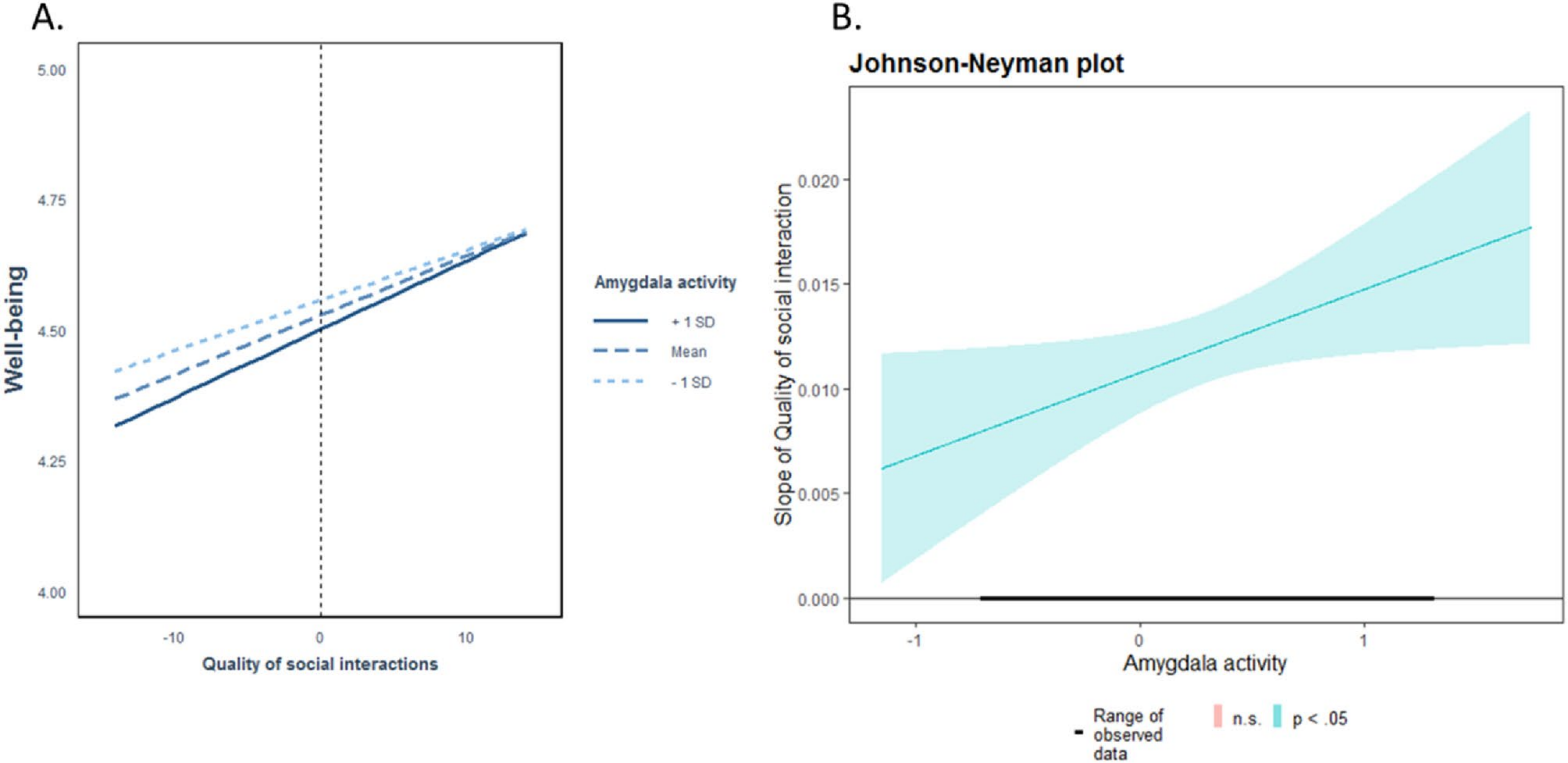
Interaction and Johnson-Neyman plots for the relationship between social interactions and well-being moderated by amygdala activity. (**A**) Interaction effect between the quality of real-life social interactions and amygdala activity on well-being. Quality of social interactions is person-mean centered and differences from zero indicate a lower or higher quality of interaction compared to the person mean. (**B**) Johnson-Neyman plots for the significant two-way interaction with amygdala activity. Johnson-Neyman plots indicate the range of observed values of a moderator (here: right amygdala activity), for which the association (i.e. ‘slope of quality of social interaction’) between quality of social interaction and well-being is significant (p < 0.05). The findings indicate that participants with higher amygdala activity during emotional processing show a stronger association between social interactions and well-being during the pandemic.

Finally, amygdala activity to negative emotional stimuli, which was assessed prior to the pandemic, was included in the model with two-way interactions to test its moderating effect. A significant interaction effect emerged between right amygdala activity and the quality of social interactions (β = 0.032, SE = 0.002, T_(2461.12)_ = 2.342, p = 0.019, Table 4), while this was not the case for left amygdala activity (β = 0.004, SE = 0.003, T_(2466.80)_ = 0.313, p = 0.754). A subsequent simple slope analysis revealed a significant moderating effect of the right amygdala, indicating a stronger effect of the quality of the most important interaction on well-being in individuals with higher amygdala activity during emotion processing (Fig. 2). These findings remained significant after including time-lagged well-being as an additional predictor of no interest (β = 0.026, SE = 0.002, T_(2406.43)_ = 2.076, p = 0.038).

**Table 4:** Mixed model effects of real-life social interactions and amygdala activity on well-being.

Predictors	Well-being				
	Estimates	Std. Beta	CI	Standardized CI	p
(Intercept)	4.6637	− 0.1183	4.0848 – 5.2426	− 0.4385 – 0.2019	< 0.001
Gender	0.1680	0.1785	− 0.2088 – 0.5449	− 0.2218 – 0.5789	0.382
Psychosocial risk factors at birth	− 0.0652	− 0.1321	− 0.1642 – 0.0338	− 0.3327 – 0.0686	0.197
Time of day	− 0.0032	− 0.0151	− 0.0110 – 0.0046	− 0.0523 – 0.0220	0.425
Critical worker status	0.2244	0.2384	− 0.1578 – 0.6066	− 0.1676 – 0.6444	0.250
Most important interaction partner	− 0.0611	− 0.0649	− 0.1415 – 0.0193	− 0.1503 – 0.0205	0.136
Quality of the relationship with the most important interaction partner	0.0011	0.0149	− 0.0013 – 0.0034	− 0.0181 – 0.0479	0.375
COVID-19-related content of the most important interaction	0.0007	0.0145	− 0.0005 – 0.0019	− 0.0118 – 0.0407	0.280
Quality of most important interaction	0.0126	0.2084	0.0106 – 0.0147	0.1808 – 0.2359	** < 0.001**
Right amygdala activity	− 0.0877	− 0.0414	− 0.5535 – 0.3781	− 0.2611 – 0.1783	0.712
Left amygdala activity	0.2150	0.0640	− 0.5384 – 0.9683	− 0.1602 – 0.2882	0.576
Right amygdala activity * Quality of most important interaction	0.0046	0.0315	0.0007 – 0.0084	0.0051 – 0.0578	**0.019**
Left amygdala activity * Quality of most important interaction	0.0010	0.0044	− 0.0054 – 0.0074	− 0.0233 – 0.0321	0.754
Weeks since lockdown	− 0.0198	− 0.0330	− 0.0888 – 0.0492	− 0.1480 – 0.0819	0.573
Random effects					
σ^2^	0.3617				
τ_00_ _Participants_	0.5722				
τ_11_ _Time of day_	0.0005				
ICC	0.60				
N	62				
Observations	2537				
Marginal R^2^ /Conditional R^2^	0.081/0.631				

Significant values are in bold.

## Discussion

The present findings revealed that engaging in positive real-life social interactions predicted higher levels of momentary well-being during the initial lockdown phase, with this relationship being dependent on the individual´s amygdala activity. In contrast, online interactions did not predict affective well-being. As such, our results emphasize an affective benefit from positive real-life social interactions during times of contact restrictions.

Contrary to our expectations, this effect was limited to real-life social interactions only, whereas online social interactions were not related to well-being. Indeed, previous studies have reported mixed findings regarding the beneficial role of online interactions in between-person designs^25,39^. For instance, in a sample of 9–12-year-olds with a Facebook profile, the real-life social network was a stronger predictor of well-being than the online social network on Facebook^40^. Moreover, while actively seeking online communication via messenger platforms is considered to foster well-being and social integration, the mere passive consumption of online social content has been linked to reduced well-being and increased perceived feelings of loneliness, often mediated by social comparison^41–44^. However, most of the studies used cross-sectional designs mainly focusing on between-subject differences, resulting in a lack of information on individual differences and in a need for within-subject designs^34,35^. While those studies were performed in adolescents prior to the pandemic^27^ and with a focus on social media platforms^26^, our design enables us differentiate the association of being in company in contrast to being alone on momentary well-being for real-life and online interactions on an individual, within-person level during times of social contact restrictions. Thereby, our findings not only suggest a superiority of face-to-face over online interactions in terms of gaining an affective benefit from social company, which is in line with previous studies in adolescents prior to the pandemic^27^, but also demonstrate that online interactions were not associated with well-being during the first lockdown at all. Given the rigorous restrictions to social life throughout the pandemic and a radical switch to online events and meetings, these findings are especially important, as they indicate that participating in a minimum of face-to-face interactions clearly fosters well-being in daily life.

In a second step, we further explored the effect of important characteristics of social interactions on well-being. Our findings highlighted that the quality of the interaction is a principal predictor of well-being, while the most important interaction partner and the relationship quality were less related to well-being. This is in line with previous findings linking positive social interactions to well-being^17,45–48^. Our findings critically extend this previous research by demonstrating that i) in fact, only the quality of the momentary interaction and not the interaction partner is an predictor, and ii) this only holds true for real-life interactions. The latter finding is especially important given that online communication has become a fundamental part of working and social environments since the outbreak of the pandemic and will likely remain so. Thus, our findings emphasize that online communication may not offer a complete substitute for the lack of social affective benefit from positive real-life interactions.

Finally, we exploratory investigated the impact of amygdala activity on the association between social interactions and momentary well-being during a global crisis, given the key role of the amygdala as a core structure in socio-emotional processing^31^ and in the relationship between social interaction and well-being^16,19,29,30,49^. Indeed, we found that amygdala activity moderated this relationship, with a stronger association observed in participants with higher amygdala activity. While previous studies have linked increased amygdala activity to heightened anxiety and diminished well-being in clinical samples^50–52^, a protective effect of preserved amygdala activation has been demonstrated for instance in resilient adults who were exposed to early life stress^53^. Thus, increased amygdala activity might in healthy individuals rather point to an adaptive responding to social stimuli for better and for worse. Indeed, participants with higher amygdala activity were more responsive to emotional information in social encounters, which thereby renders them more sensitive to high and low quality interactions.

Some limitations of the present study need to be addressed. Given the longitudinal design of our study and due to the restricted time frame for data collection, only a quarter of our initial sample was able to take part in this follow-up assessment, resulting in a relatively small sample size, particularly for the interaction analysis, and thereby warranting a validation of our results in a larger cohort. Moreover, given that findings on test–retest validity of amygdala activity during emotional tasks are heterogeneous with moderate effects at best (for instance, Ref.^54^), definite statements on the ecological validity of amygdala activation are premature. However, those who took part did not systemically differ from the initial sample in terms of psychosocial risk factors at birth or gender distribution. In addition, while our sample represents the German general population within this age range very well (see sample description), children and adolescents might show a different pattern of engaging and using online interactions (for instance via online gaming). Therefore, generalizability of our results to other age groups, especially adolescents, might be limited. Moreover, while we were interested in the effect of social interactions and their specific characteristics (e.g., the social interaction quality) on current mood, we acknowledge the possibility that this relationship might also turn out to be bidirectional. Therefore, further studies are needed to disentangle those interaction effects.

Taken together, our results highlight the affective benefit of social contacts during the initial lockdown of the COVID-19 pandemic, when access to familiar social activities was limited. Importantly, we showed that positive real-life social interactions predicted increased momentary well-being, whereas participating in online communication was not related to current mood levels. Finally, this relationship was dependent on the level of amygdala activation, with the highest responsiveness to interaction quality found in individuals with the highest activity.

## Methods

### Sample

The present investigation was conducted within the Mannheim Study of Children at Risk, an ongoing longitudinal study of the long-term outcomes of early psychosocial and biological risk factors following participants since birth^55^. The initial sample consisted of 384 children born between 1986 and 1988 in the Rhine-Neckar region of Germany. Infants were recruited from two obstetric and six children’s hospitals and were included in the sample according to a two-factorial design intended to enrich and control the risk status of the sample. Participants were primarily of Caucasian ethnicity (99%)*.*

Starting at the age of 3 months, information on mental health, stressful life events and sociodemographic status was collected prospectively, up to the most recent assessment wave at the age of 32 to 33 years. This assessment wave started in early 2019 and was disrupted by the COVID-19 pandemic in mid-March. So far, 240 participants from the initial sample have agreed to take part in this assessment wave. The whole assessment wave consisted of a comprehensive questionnaire package on physical and mental health, a diagnostic interview, a magnetic resonance imaging (MRI) session, and an EMA. Up to the start of the first lockdown on March 22nd 2020, 165 participants had completed all parts of the assessment wave. Starting shortly after the lockdown in Germany in April 2020, participants who had completed the whole procedure were invited to take part in a short online survey and to repeat the EMA procedures during the COVID-19 pandemic. A total of 133 participants completed the online survey, of whom 75 agreed to take part in another EMA measurement. Only full datasets, consisting of functional MRI and EMA data, were considered in the present study, resulting in a total of 62 participants (58% female; distribution in the current sample: 21 (33.9%) participants without psychosocial risk at birth, 21 (33.9%) with low psychosocial risk, and 20 (32.2%) with high psychosocial risk). Moreover, participants included in the present study did not systematically differ from the dropout sample with regard to IQ (T _(130)_ = 1.054, p = 0.294), income (T_(127)_ = 0.442, p = 0.660) or educational level (T _(129)_ = − 1.238, p = 0.218). In addition, when comparing our participants to publicly available reference data from the German general population, our participants show similar educational levels (current sample: highest educational degree (university) = 24.2%; German reference sample = 28.6%), and report a comparable household financial situation (current sample: mean monthly income = 3957 €; German reference sample: mean monthly income = 3580 €^56^).

The study was approved by the Ethics Committee of the University of Heidelberg, Germany (protocol no. 2015-612N-MA), written informed consent was obtained from all participants, and participants were financially compensated. All procedures involving human participants were performed in accordance with the Declaration of Helsinki.

### EMA procedures

Starting in April 2020, participants were asked to install a commercial e-diary app (MovisensXS, version 1.4.3) on their own Android smartphone. After an extensive briefing on the use of the e-diary app, they were asked to carry the smartphone with them for one week. Participants were encouraged to answer at least 80% of all prompts, which was carefully observed throughout the measurement. This approach enabled us to offer immediate support if necessary to ensure a sufficient compliance rate, given that a-priori power calculation was not performed due to both the immediate start of the assessment and the limited accessibility of our participants. Moreover, participants were carefully instructed to avoid responding to the prompts in potentially dangerous situations.

The e-diary began on a fixed date depending on the participants´ availability. Prompts were scheduled between 8 am and 10 pm at fixed intervals of 120 min, resulting in eight triggers per day and 56 triggers per week. To answer a single prompt, participants had to actively accept the prompt. Then, they were presented with a questionnaire, which took them approximately 90 s to complete. Participants also had the possibility to postpone a prompt for a maximum of 25 min.

#### Well-being

Well-being was measured using a short version of the German adaptation of the Positive and Negative Affect Schedule (PANAS)^57^ with additional items capturing stress reactivity^58–61^. Participants were asked to rate their current positive feelings on a 7-point Likert scale (1 = fully disagree, 7 = fully agree). Mean scores for positive affect were calculated for each prompt and used as a dependent variable in all analyses. Between- and within-person reliability coefficients for positive affect (R_kf_ = 0.99; R_cn_ = 0.62) were calculated using mixed models^62^ and ranged from moderate to high.

#### Social interactions

Participants were asked about their engagement in real-life and online social interactions separately. Real-life social interactions were defined as interactions in which the participants interacted face to face with another person, whereas online interactions were defined as all interactions with technological assistance, i.e. via a smartphone or computer. Participants were asked to report whether they had engaged in real-life and online interactions within the last two hours before the prompt (i.e. the interval between two prompts). Next, they reported with whom they had their most important interaction by choosing from a single-choice selection consisting of family members, life partner, friend, colleague, supervisor, stranger, or a pet. Finally, on a visual analogue scale ranging from 0 to 100, participants indicated how much they liked the previously selected person (0 = not at all, 100 = very much), whether this interaction was COVID-19-related (0 = not at all, 100 = very much), and how this interaction was experienced (0 = very negative, 100 = very positive). If participants reported no interactions within the current time frame, no follow-up questions were presented (see the method section of the Supplementary Information for the full EMA questionnaire).

### Amygdala activity

#### Task

Amygdala activity was derived from a modified version of an emotion regulation task, which was completed within the regular assessment wave at the age of 32 to 33 years before the COVID-19 pandemic. Details of the task have been published elsewhere^63,64^. In brief, participants were asked to watch aversive (‘Look negative’) or neutral (‘Look neutral’) pictures or to reappraise negative (‘Reappraisal’ condition) pictures from the International Affective Picture System (IAPS)^65^. In the reappraisal condition, participants were asked to use the cognitive strategy of reappraisal to decrease the intensity of their negative affect. During the look negative and neutral conditions, participants were instructed to simply watch the depicted scenarios without actively changing their emotional state evoked by the pictures.

The fMRI task consisted of a randomized block-related design, in which every block started with a 5 s presentation of the instruction form (i.e. ‘Look’ or ‘Reappraise’). Subsequently, participants viewed either four negative or four neutral pictures for 5 s each according to the presented condition. Immediately thereafter, participants were asked to rate the intensity of currently perceived negative feelings on a 7-point Likert scale (1 = no negative feelings at all; 7 = extremely negative feelings) via button press. Each block was interspersed with a 3 s inter-trial interval. The total task consisted of three blocks per condition (12 blocks in total) and lasted for 6 min 37 s.

#### Functional MRI data acquisition and preprocessing

Functional MRI data collection consisted of a localizer scan followed by a blood oxygen level-dependent (BOLD)-sensitive T2*-weighted echo-planar imaging (EPI) sequence and structural T1-weighted sequence using a 3 T scanner (PrismaFit; Siemens) with a standard 32-channel head coil. For functional imaging, a total of 186 volumes with 36 slices covering the whole brain (matrix 64 × 64, resolution 3.0 × 3.0 × 3.0 mm with 1 mm gap, repetition time = 2100 ms, echo time = 35 ms, flip angle = 90°) were acquired for each task. The slices were inclined 20° from the anterior/posterior commissure level. The first 11 images were discarded to allow longitudinal magnetization to reach equilibrium.

#### Functional MRI data analyses

Statistical Parametric Mapping (SPM 12) implemented in MATLAB R2017b was used to analyze functional data. Preprocessing included slice time correction, realignment, co-registration, spatial normalization and spatial smoothing. At the individual subject first-level analysis, onsets and durations of each block were convolved with the SPM 12 canonical hemodynamic response. As we were interested in the amygdala activity during an emotional experience of aversive stimuli, only first-level contrast images for ‘Look negative’ > ‘Look neutral’ were created. For the group-level analysis, individual contrast images of ‘Look negative’ > ‘Look neutral’ condition were entered into a random-effects analysis. Wake Forest University (WFU) PickAtlas^66^ was used to generate bilateral amygdala region of interest (ROI) masks. The Region of interest Extraction (REX, version 2.1) toolbox^67^ was applied to extract mean ROI activity values for both left and right amygdala activity. Robust bilateral amygdala activation for the contrast ‘Look negative’ > ‘Look neutral’ (left amygdala: t_(61)_ = 4.25, p_FWE_ = 0.001, k_E_ = 32; right amygdala: t_(61)_ = 3.48, p_FWE_ = 0.004, k_E_ = 26) was obtained.

### Covariates

Psychosocial risk factors at birth, gender, time of day, critical worker status (yes/no), and number of weeks since social contact restrictions began (22nd March 2020) were included as covariates of no interest. Psychosocial risk factors at birth were included as a covariate of no interest to control for the possible detrimental impact of environmental risk factors on subjective well-being and mood^68^. Psychosocial risk was assessed using a standardized interview according to an enriched family adversity index 47 at the participants’ age of 3 months, covering 11 items of the family environment, the parents, and their partnership (e.g. parental psychiatric disorders, overcrowding, or ongoing parental conflicts). A sum score of psychosocial risks were calculated by adding up the presence of all items. The number of weeks since the social contact restrictions began was included as an additional covariate of no interest to limit the possible habituation effects regarding the contact ban and to control for the impact of continuous loosening of existing restrictions.

### Data analysis

Separate multilevel analyses were conducted to analyze the association between social interactions and current well-being. For each model, EMA data of current well-being as dependent variable and dimensions of social interactions as predictor variables (level 1) were nested within participants (level 2). For all models, level-1 predictors of interest (i.e. characteristics of real-life and online social interactions) were person-mean centered, whereas level-2 variables (amygdala activity) were grand-mean centered. Furthermore, covariates of no interest (gender, time of day, critical worker status, number of weeks since restrictions began, and psychosocial risk factors) were entered in all models. Psychosocial risk factors were grand-mean centered whereas time of day was calculated in minutes by subtracting the daily start time (i.e. 8 am) from all values. Finally, random coefficient models were created, with random effects for the intercept, fixed effects for level-1 predictors, and random slope effects for time of day.

In a first step, we explored whether the presence (yes/no) of real-life and online social interactions was associated with well-being (Model I). Next, we further investigated only those time points for which previous social interactions were reported. Therefore, we explored whether the quality of a social interaction, the interaction partner (family member vs. non-family member), or the liking of the interaction partner were related to well-being (Model II). Finally, we included amygdala activity (Model III) to the previous models to test for its moderating effect. Therefore, two-way interactions for significant level-1 predictors were calculated and added to the model. To control for possible dependencies of previous mood states on the following mood state, additional sensitivity analyses were calculated by including time-lagged data of positive affect (t + 1) as an additional predictor of no-interest into the model.

All multilevel models were analyzed with the R packages lme4^69^ and lmerTest^70^ to compute p-values. To further analyze the interaction terms, simple slope analyses and Johnson-Neyman plots and intervals were computed^71,72^.

Granger causality tests were performed to analyze a possible causal relationship between characteristics of social interactions and well-being. Therefore, we used well-being as dependent variable (first time series) and characteristics of social interaction as predictor variable (second time series) and vice versa. The default setting of one lag was used. All analyses were performed using the R package lmtest^73^.

## Supplementary Information

Supplementary Information.

## Data Availability

The data that support the findings of this study are available from the corresponding author, N.E.H., upon reasonable request.
