# Supplementary material for: The importance of high quality real-life social interactions during the COVID-19 pandemic
Source: Sci Rep. 2023 Mar 4;13:3675. doi: 10.1038/s41598-023-30803-9 (PMC9985477; doi:10.1038/s41598-023-30803-9)
Supplement: Supplementary file 1 — Supplementary Information. [file 41598_2023_30803_MOESM1_ESM.docx]

**The importance of high quality real-life social interactions during the COVID-19 pandemic**

Maximilian Monninger^1^, Pascal-M. Aggensteiner^1^, Tania M. Pollok^1^, Anna Kaiser^1^, Iris Reinhard^2^, Andrea Hermann^3-5^, Markus Reichert^6,7^, Ulrich W. Ebner-Priemer^6,7^, Andreas Meyer-Lindenberg^6^, Daniel Brandeis^1, 8,9^, Tobias Banaschewski^1^, Nathalie E. Holz^1,10,11,12*^

# **Results**

## Supplementary Table 1: Mixed model effects for distinct characteristics of online social interactions and well-being

|  | **Well-being** | | | | |
| --- | --- | --- | --- | --- | --- |
| ***Predictors*** | ***Estimates*** | ***std. Beta*** | ***CI*** | ***standardized CI*** | ***p*** |
| (Intercept) | 4.4454 | -0.1614 | 3.8190 – 5.0718 | -0.4631 – 0.1403 | **<0.001** |
| Gender | 0.1645 | 0.1684 | -0.2091 – 0.5382 | -0.2140 – 0.5508 | 0.388 |
| Psychosocial risk factors at birth | -0.0803 | -0.1541 | -0.1786 – 0.0180 | -0.3427 – 0.0346 | 0.109 |
| Daytime | 0.0026 | 0.0115 | -0.0082 – 0.0133 | -0.0363 – 0.0592 | 0.638 |
| Critical worker status | 0.1904 | 0.1948 | -0.1888 – 0.5695 | -0.1932 – 0.5829 | 0.325 |
| Most important online interaction partner | -0.1111 | -0.1137 | -0.1911 – -0.0311 | -0.1956 – -0.0319 | **0.006** |
| Quality of the relationship to the most  important online interaction partner | 0.0039 | 0.0511 | 0.0010 – 0.0067 | 0.0137 – 0.0884 | **0.007** |
| COVID-19-related content of the most important online interaction | 0.0006 | 0.0151 | -0.0006 – 0.0018 | -0.0163 – 0.0465 | 0.347 |
| Quality of the most important online interaction | 0.0052 | 0.0769 | 0.0029 – 0.0075 | 0.0425 – 0.1113 | **<0.001** |
| Weeks since Lockdown | 0.0008 | 0.0013 | -0.0784 – 0.0801 | -0.1214 – 0.1240 | 0.983 |
| **Random Effects** | | | | | |
| σ^2^ | 0.43 | | | | |
| τ_00_ _Participants_ | 0.67 | | | | |
| τ_11_ _Time of day_ | 0.00 | | | | |
| ICC | 0.56 | | | | |
| N | 62 | | | | |
| Observations | 1849 | | | | |
| Marginal R^2^ / Conditional R^2^ | 0.041 / 0.581 | | | | |

## Quality of online social interactions and well-being (Model IIb)

In situations, in which online social interactions were reported (N= 1849), we found significant associations for the most important online interaction partner (p= 0.006), liking of the most important online interaction partner (p = 0.007), and the quality of online social interactions (p < 0.001), and well-being (Supplementary Table 1). While the quality of online social interactions and liking of the most important online interaction partner predicted increased momentary well-being, and affective benefit was only observed in online communication with non-family members. Only the quality of the most important online interaction remained significant after including time-lagged well-being as an additional predictor of no interest (p < 0.001).

**Amygdala activity, online social interactions, and well-being (Model IIIb)**

There was no significant interaction for the left or right amygdala and the quality of online social interactions (left: p = 0.753; right: p = 0.567).

# **Methods**

## EMA Questionnaire

1. At the moment, I feel … happy, lonely, pleased, sad, unsure, anxious, enthusiastic, energetic, depressed, guilty, relaxed, glum, content, leery, irritable strongly disagree - strongly agree (1-7)
2. Please remember the most important negative event since the last prompt. How did you feel? No event - very negative (0-100).
3. Please remember the most important negative event since the last prompt. How did you feel? No event - very positive (0-100)
4. How many real-life contacts did you have since the last prompt?
5. Please remember the most important interaction. With whom did you have contact?
6. Please remember the most important interaction. Did you like this person? Absolutely not - neutral - very much (0-100)
7. Please remember the most important interaction. How did you experience this interaction? Very negative - neutral - very positive (0-100)
8. Please remember the most important interaction. Was this interaction related to the COVID-19 pandemic? Not all - very much (0-100)
9. How many digital contacts did you have since the last prompt?
10. Please remember the most important interaction. With whom did you have contact?
11. Please remember the most important interaction. Did you like this person? Absolutely not - neutral - very much (0-100)
12. Please remember the most important interaction. How did you experience this interaction? Very negative - neutral - very positive (0-100)
13. Please remember the most important interaction. Was this interaction related to the COVID-19 pandemic? Not all - very much (0-100)
